# An Objective Approach to Determining the Weight Ranges of Prey Preferred by and Accessible to the Five Large African Carnivores

**DOI:** 10.1371/journal.pone.0101054

**Published:** 2014-07-02

**Authors:** Hayley S. Clements, Craig J. Tambling, Matt W. Hayward, Graham I. H. Kerley

**Affiliations:** 1 Centre for African Conservation Ecology, Department of Zoology, Nelson Mandela Metropolitan University, Port Elizabeth, South Africa; 2 School of Environment, Natural Resources & Geography and School of Biological Science, Bangor University, Gwynedd, United Kingdom; Centre for Ecological and Evolutionary Studies, Netherlands

## Abstract

Broad-scale models describing predator prey preferences serve as useful departure points for understanding predator-prey interactions at finer scales. Previous analyses used a subjective approach to identify prey weight preferences of the five large African carnivores, hence their accuracy is questionable. This study uses a segmented model of prey weight versus prey preference to objectively quantify the prey weight preferences of the five large African carnivores. Based on simulations of known predator prey preference, for prey species sample sizes above 32 the segmented model approach detects up to four known changes in prey weight preference (represented by model break-points) with high rates of detection (75% to 100% of simulations, depending on number of break-points) and accuracy (within 1.3±4.0 to 2.7±4.4 of known break-point). When applied to the five large African carnivores, using carnivore diet information from across Africa, the model detected weight ranges of prey that are preferred, killed relative to their abundance, and avoided by each carnivore. Prey in the weight ranges preferred and killed relative to their abundance are together termed “accessible prey”. Accessible prey weight ranges were found to be 14–135 kg for cheetah *Acinonyx jubatus*, 1–45 kg for leopard *Panthera pardus*, 32–632 kg for lion *Panthera leo*, 15–1600 kg for spotted hyaena *Crocuta crocuta* and 10–289 kg for wild dog *Lycaon pictus*. An assessment of carnivore diets throughout Africa found these accessible prey weight ranges include 88±2% (cheetah), 82±3% (leopard), 81±2% (lion), 97±2% (spotted hyaena) and 96±2% (wild dog) of kills. These descriptions of prey weight preferences therefore contribute to our understanding of the diet spectrum of the five large African carnivores. Where datasets meet the minimum sample size requirements, the segmented model approach provides a means of determining, and comparing, the prey weight range preferences of any carnivore species.

## Introduction

Carnivore numbers are declining globally, with a reduction in distribution and abundance leading to almost a quarter of species now being threatened with extinction [1]. Since predator populations are limited by available food [2], a major consideration for large carnivore conservation should be an adequate abundance of suitable prey [3], [4]. Ensuring suitable prey availability is dependent on an understanding of which prey are killed by the predator, and how this relates to prey availability.

The concept of prey preference is useful in identifying which prey are likely to be targeted by a predator, as it identifies prey which comprise a greater proportion of a predator’s diet than expected according to the prey item’s relative abundance in the prey community [5]. Recent reviews on carnivore feeding ecology have employed multi-site diet analyses in order to ascertain which prey species and prey weight ranges are consistently preferred by each of the five large African carnivores: cheetah *Acinonyx jubatus,* leopard *Panthera pardus*, lion *Panthera leo,* spotted hyaena *Crocuta crocuta* and African wild dog *Lycaon pictus* [5]–[9]. However, the method employed by these studies to determine preferred prey weight ranges is subjective. For each carnivore a distance-weighted-least-squares curve was fitted to a plot of prey mass versus a measure of prey preference (the Jacobs’ Index; [10]). The weight range representing the peak of this curve was identified as the “most preferred” weight range by linking positive Jacobs’ Index values to prey body mass values on the x-axis [5]. However, this approach does not offer a statistically independent means of determining where on the peak of the curve the “most preferred” weight range lies, and is therefore subjective. Furthermore, extracted values may misrepresent actual preferred prey weight ranges if the coefficient of determination of the model fit is low, indicating that outlying values are influencing model fit (e.g. wild dog; [9]). The accuracy of this method is therefore questionable, and it is neither replicable nor comparable across carnivore species.

Despite the questionable accuracy of these weight ranges, they are serving as a point of departure for a wide range of applications, including ecological studies [11], [12], conservation suggestions [13] and human-wildlife conflict issues [14]. Given the importance of providing accurate descriptions of carnivore diet and prey preference, we present a novel and objective approach for determining a predator’s preferred prey weight range, which we apply to the preference data from the original carnivore prey preference papers in order to recalculate the previously subjective preferred prey weight ranges. Furthermore, our novel approach allows for the identification of not only the preferred prey weight range (as did the previous method), but also the weight range of prey killed relative to their abundance in the prey community, and the weight range of prey avoided by the carnivore. This approach is shown to allow for a more complete description of each predator’s prey weight spectrum.

## Methods

### Prey preference calculation

Commonly used preference indices such as the forage ratio and Ivlev’s electivity index [15] suffer from non-linearity, bias towards rare food items, increasing confidence intervals with increasing heterogeneity, being unbound or undefined and lacking symmetry between selected and rejected values [10]. While other preference ratings have also been developed (e.g. [16]), none are without bias to some extent (such as bias toward rare food items; [16] and, consistent with previous prey preference studies for the five large African carnivores [5], we use the Jacobs’ Index (J.I.; [10]) which minimizes the above-listed problems:
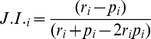



J.I. standardizes the relationship between a prey species’ relative abundance at a site, *p_i_* (i.e. the proportion *p* that prey species *i* makes up of the total abundance of censused prey at a site) and the proportion of carnivore kills that prey species *i* comprises, *r_i_,* to a value between +1 and −1. The literature was reviewed by Hayward and colleagues [5]–[9] for studies describing the diet of the five large African carnivores, as well as corresponding measures of prey availability. The kill data collected in these studies were derived from both incidental observations and continuous follows, as well as from scat analyses in the case of leopard and spotted hyaena. While incidental observations are biased toward larger prey; this bias against smaller items is generally alleviated by the undercounting of small prey species in aerial counts [5]. The current study focussed on the diet of each carnivore in Africa and therefore omitted the datasets from Asia included in the previous diet analyses of lion and leopard. The sources, location and timing of African studies used by Hayward and colleagues and the number of kills recorded in each are presented in Table S1. For each carnivore, prey abundance and kill data were used to calculate a J.I. value for each listed prey species at each site detailed in Table S1, as done in the original preference papers. Each potential prey species was allocated a standard species mass of three-quarters of the mean adult female body mass [17], in order to account for calves and sub-adults eaten ([5], [18]; Table S2). This assumption appears robust when tested with kill data for leopard [19].

### Segmented model approach to calculating preferred prey weight ranges

**Figure 1:**
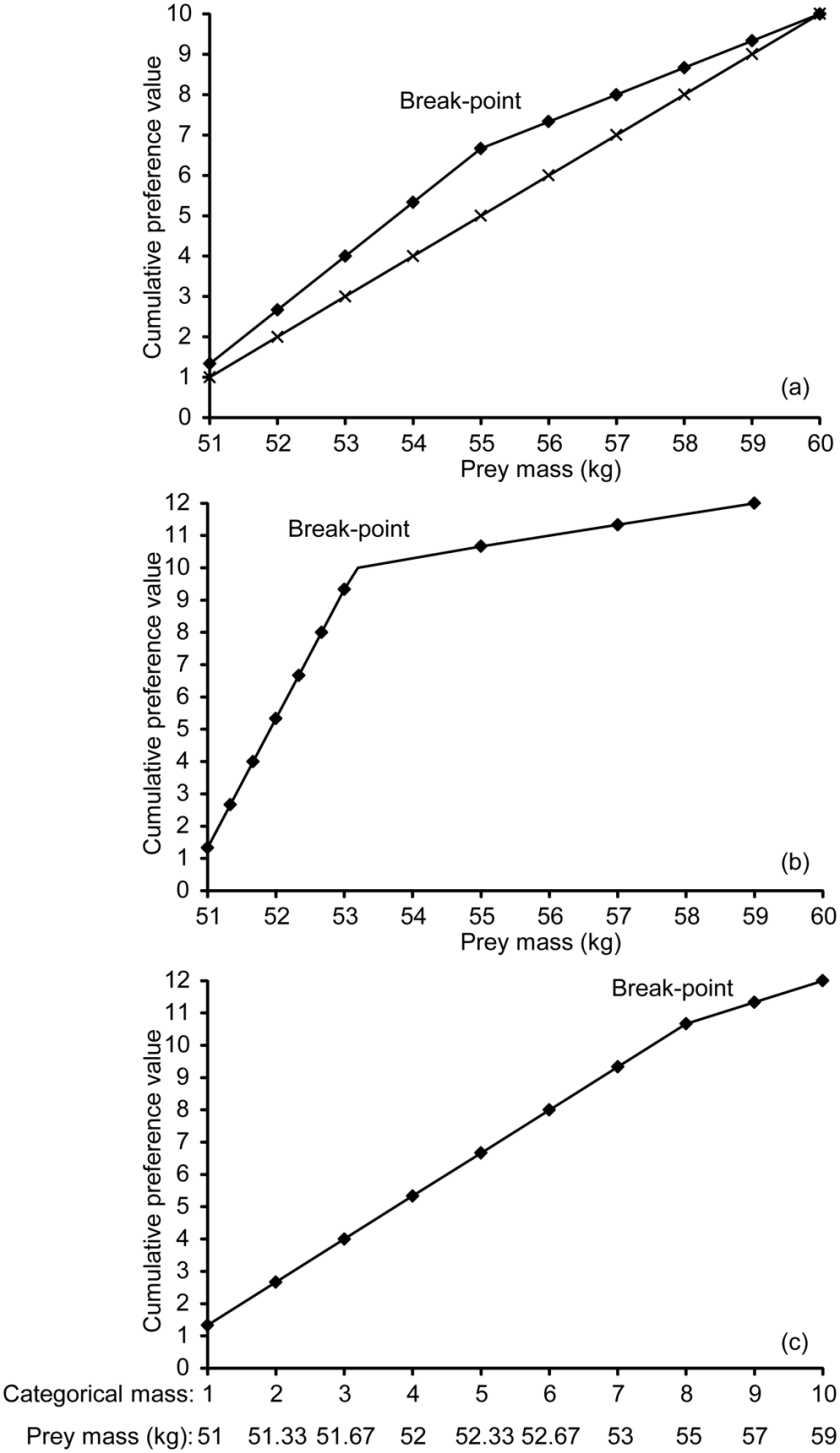
Relationships between prey mass and prey preference for hypothetical predator-prey communities. In (a) prey masses are evenly distributed and one predator prefers prey of all masses equally (crosses), and another predator prefers prey weighing 51 to 55 kg twice as much as those weighing more than 55 kg (diamonds). In (b) prey masses are not evenly distributed and the predator prefers prey weighing 51 to 55 kg twice as much as those weighing more than 55 kg. In this example the break-point is incorrectly detected at 53.15 kg instead of at 55 kg. In (c) prey masses are represented by categorical values to generate even mass distribution and the predator prefers prey weighing 51 to 55 kg twice as much as those weighing more than 55 kg. In this example the break-point is correctly detected at 55 kg.

To explain our approach for determining the preferred prey weight range of each large carnivore, we use two hypothetical predators which have 10 prey species available to them, each differing in weight by 1 kg and weighing between 51 kg and 60 kg. First we consider a situation in which a predator displays equal preference for all 10 species. If the mass of each prey species was regressed against a measure of prey preference (or “preference value”) for that species plus the preference values of all smaller species, this regression would be positive and linear (Fig. 1a - crosses). If a predator preferred the species weighing between 51 and 55 kg twice as much as the species weighing more than 55 kg, the slope of the relationship between prey mass and cumulative preference would be twice as steep for the first five prey masses than for the second five (Fig. 1a - diamonds). In this case the relationship between the response and explanatory variables would be piecewise linear (segmented), represented by two straight lines connected at a “break-point” [20]. For the hypothetical predator preferring smaller prey, the break-point or change in preference is known to occur between 55 kg and 56 kg, by definition of the assumptions set out above. Using this approach for an actual predator whose prey weight preferences are unknown, a segmented model can be used to detect break-points at which the relationship between prey mass and prey preference changes significantly, thus identifying weight ranges of prey that differ in terms of predator preference.

While in our first hypothetical example prey weights are evenly distributed (in increments of 1 kg), this is unlikely to be the norm in real prey communities. Let us again consider a hypothetical predator which has ten prey species weighing between 51 and 60 kg available to it, and which prefers those weighing 51 to 55 kg twice as much as those weighing more than 55 kg. Consider a prey community in which the prey masses are not evenly distributed, with seven of the ten species weighing 53 kg or less. In such a situation, break-points representing changes in slope do not reflect changes in preference alone (as intended), but are confounded by prey mass groupings which influence the slope of the relationship (Fig. 1b). In order to control for this, prey species masses can be ranked from lightest to heaviest and converted into categorical integer values of equal increment (from 1 to 10), in order to ensure that all masses have equal weighting. When this transformation is implemented for the unevenly distributed masses of prey weighing between 51 and 60 kg, the biases in slope and detected break-point (caused by uneven prey mass distribution) are eliminated and the break-point correctly detects the change in predator prey preference at a prey mass of 55 kg (Fig. 1c).

### Assessing the accuracy of the segmented model approach

In order to assess the accuracy of the proposed segmented model approach in determining a predator’s prey weight preferences, and to determine minimum sample size requirements, we simulated predator diets with known changes in prey preference (and thus break-points). We simulated prey preference datasets with one, two, three and four known changes in preference, as four was the maximum number of break-points detected for the five large African carnivores (see results). Predator prey preference was simulated by randomly generating between two (for one change in preference) and five (for four changes in preference) sets of J.I. values. The first and fifth (where relevant) sets of J.I. values were randomly generated between −1 and −0.3; the second and fourth (where relevant) sets with J.I. values between −0.3 and 0.3 and the third (where relevant) set with J.I. values between 0.3 and 0.85 (while +1 exists in theory, it does not in practice). These sets represent prey groups that are avoided (first and fifth set), killed relative to their abundance (second and fourth set) and preferred (third set; confirmed using t-tests: see *Calculating the predator’s preference for identified weight ranges* section below). Changes in preference, and thus break-points in the relationships, therefore exist between these prey groups. For each simulation, we randomly selected a total prey species sample size between six and 100, and these species were randomly allocated between the preference groups (between two and five preference groups). For each break-point assessment (one to four breaks) we conducted 1000 simulations. For each simulation, we assigned a categorical integer value to each prey species, commencing at 1 for the first randomly generated species in the first group and ending with the last randomly generated species in the last group. We fitted a segmented model to the relationship between prey preference (cumulative J.I.+1 to ensure non-negative values) and prey categorical value and determined how many break-points were detected and at what prey categorical value they were detected. We calculated the percentage of simulations in which the correct number of break-points was detected and related this to the minimum number of species occurring within a preference group. The ability of the segmented model to detect all break-points increased with an increase in the minimum number of species within any preference group. Therefore, the accuracy of break-point detection can be assessed as a function of the total minimum sample size, determined by multiplying the minimum sample in the smallest preference group by the number of groups. Break-point detection accuracy was assessed by calculating, for each simulation which met the minimum sample size requirements, the absolute difference in prey species categorical value between the known break-point and the detected break-point. These models were conducted in the open source statistical package R (R Development Core Team 2012) using the segmented generalized linear model function in the “segmented package” ([21]; see example code detailed in Code S1). The segmented model approach was performed for each of the five large carnivores (separately), using the data transformations detailed below.

### Large carnivore prey preference assessment: Prey mass (x axis) data transformation

The standard species masses of all listed prey species were not evenly distributed, as a result of a preponderance of prey species with lower body masses compared to higher body masses (Table S2). Prey species (for which J.I. values were available from two or more sites) were therefore ranked from lightest to heaviest according to standard species masses and each species was allocated an integer value, commencing at 1 for the lowest prey mass (Table S2). These rank values are hereafter referred to as prey mass-ranks.

### Large carnivore prey preference assessment: Prey preference (y axis) data transformation

A mean J.I. value was calculated for prey species with a J.I. value for two or more sites ([5]; Table S2). The mean J.I. value for each prey species was standardized (+1 to ensure non-negative values) and used as an index of prey preference. Standardized mean J.I. values are hereafter referred to as J.I.+1 values. For each prey species, a corresponding cumulative J.I.+1 value was calculated, commencing at the prey species with a mass-rank of 1.

### Large carnivore prey preference assessment: Fitting the segmented model

Prey mass-ranks were plotted against corresponding cumulative J.I.+1 values and a segmented model was fitted to these plots. This approach requires the number of break-points in the model to be stipulated before the model is run [21]. The optimum number of break-points (where more than one existed) was therefore selected using Akaike’s information criterion (AIC; [22]). Straight line and best-fit polynomial functions were also fitted to the data and Akaike’s information criteria used to ascertain whether the segmented model was the best-fit function.

### Calculating the large carnivore’s preference for identified weight ranges

As a segmented model was the best-fit function for all five carnivores, mass-ranks at which the best-fit model detected break-points were then translated back into actual prey masses (standard species masses; Table S2). Where a break-point fell directly on a mass-rank, the species mass corresponding to that mass-rank was included in the weight range to the left of this break-point. Where a break-point did not fall exactly on a mass-rank but rather between two mass-ranks, the corresponding species mass between the two adjacent species masses was determined. Within each of the weight ranges identified by model break-points, the actual degree of prey preference was quantified. This was done by calculating the proportion of the total carnivore kills (*r_i_*) and censused prey community (*p_i_*) in each prey weight range *i* at each site listed in Table S1. From these values a J.I. value for each weight range was calculated for each site. The mean J.I. value of each prey weight range across sites was tested for significant preference or avoidance using a single sample t-test against a mean of zero where data conformed to the assumptions of normality, and a Wilcoxon signed-rank test where data did not [23]. A mean J.I. value significantly greater than zero indicated a preferred prey weight range, a mean J.I. value not significantly different from zero indicated prey in a weight range killed relative to their abundance and a mean J.I. value significantly less than zero indicated an avoided prey weight range. Preferred prey weight ranges were compared with those determined by [5]–[9].

### The percentage of large carnivore diet included in prey weight ranges at test sites

In order to test whether the segmented model method accurately described each carnivore’s prey weight spectrum as hypothesized; the literature was reviewed for additional descriptions of carnivore diet in Africa which were not used to develop the segmented models. These sites were then used as independent test sites (Table S3). For each of these sites, we calculated the proportion of kills which fell within the weight ranges of prey identified by this study as (i) preferred and (ii) killed relative to their abundance. The weight range which encompasses both (i) and (ii) is hereafter referred to as the “accessible” prey weight range. The mean proportion of kills in the preferred and accessible prey weight ranges across sites was determined for each large carnivore. We tested whether the mean proportion of kills in the preferred weight range was significantly different from that in the accessible prey weight range for each large carnivore, using a paired t-test [23]. Statistical analyses were performed in the statistical package R, at a significance level of 0.05.

## Results

The accuracy of detecting the correct number of changes in a predator’s prey weight preference increases with increasing prey species sample size (Table 1). A greater sample size of prey species is needed to detect a greater number of changes in a predator’s prey weight preferences (Table 1). To have a 75% chance of detecting all known break-points, the minimum prey species sample sizes are 30, 32, 9 and 6, to detect 4, 3, 2 and 1 break-points, respectively (Table 1). At these sample sizes, detected break-points fall, on average, within 2.7, 1.6, 1.1 and 1.3 mass-ranks of the known break-point, respectively (Table 1).

**Table 1:** Minimum species sample size at which all known break-points in prey preference were detected in 75% to 100% of simulations; and the mean (±SD) absolute difference in prey category between known break-points and detected break-points at each minimum sample size.

Percent of simulations	Minimum sample size (mean ± SD error in detected break-point)			
	4 break-points	3 break-points	2 break-points	1 break-point
100	65 (1.4±3.6)	60 (0.6±0.2)	48 (0.7±0.4)	24 (0.7±0.6)
95	65 (1.4±3.6)	60 (0.6±0.2)	30 (1.1±3.5)	20 (0.8±0.7)
90	60 (1.1±3.0)	56 (0.6±0.3)	24 (1.1±3.4)	14 (0.8±2.2)
85	45 (1.9±4.0)	48 (0.6±0.3)	21 (1.1±3.4)	8 (1.1±3.3)
80	45 (1.9±4.0)	48 (0.6±0.3)	12 (1.1±4.4)	6 (1.3±4.0)
75	30 (2.7±4.4)	32 (1.6±3.7)	9 (1.1±4.6)	6 (1.3±4.0)

**Figure 2:**
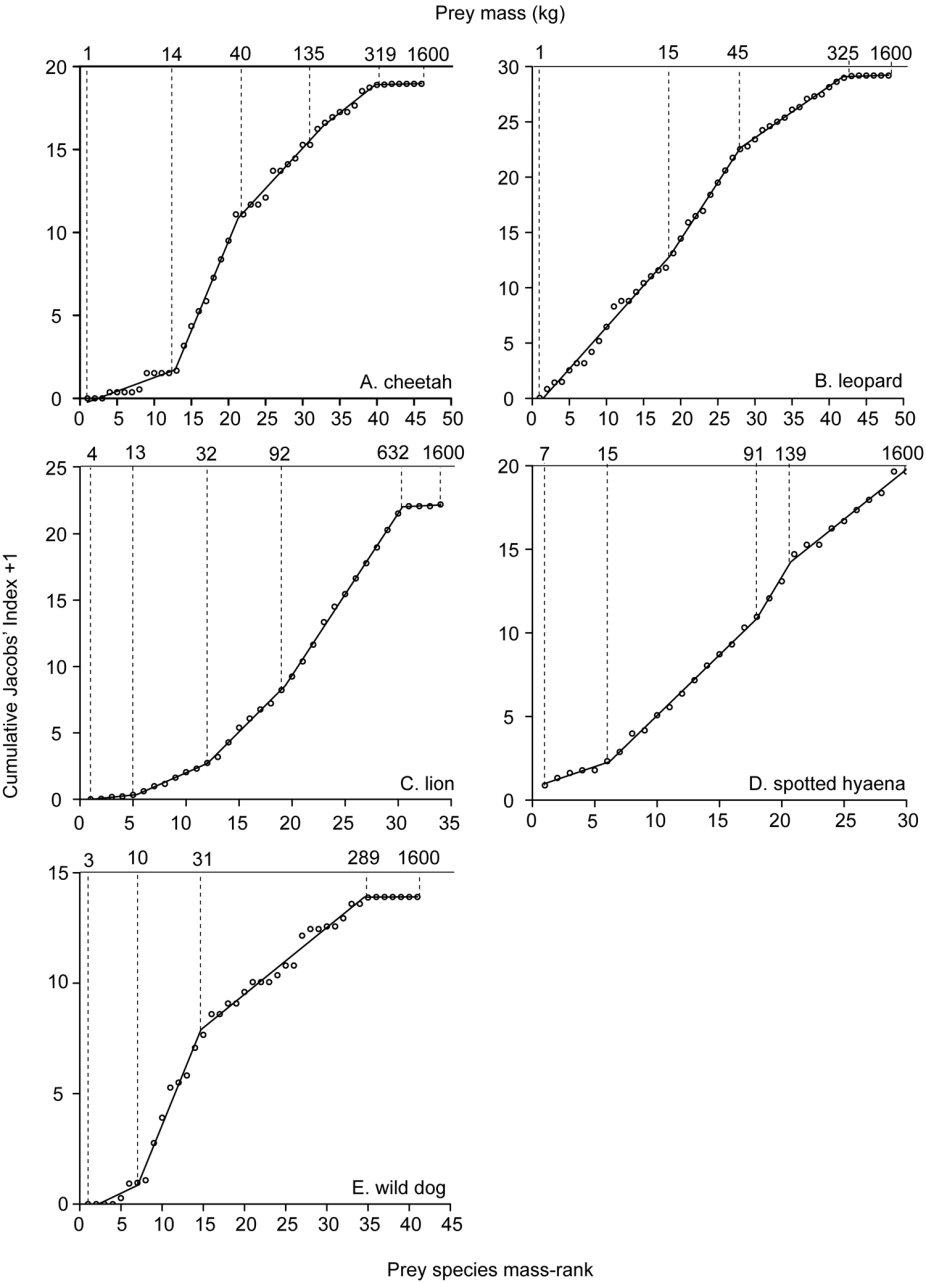
Segmented models of the relationship between the mass-rank of each prey species and carnivore preference; for cheetah, leopard, lion, spotted hyaena and wild dog. Preference is indicated by cumulative Jacobs’ index+1 values. Actual prey species’ masses which correspond to the lowest, break-point, and highest prey mass-ranks are indicated above the figures.

**Figure 3:**
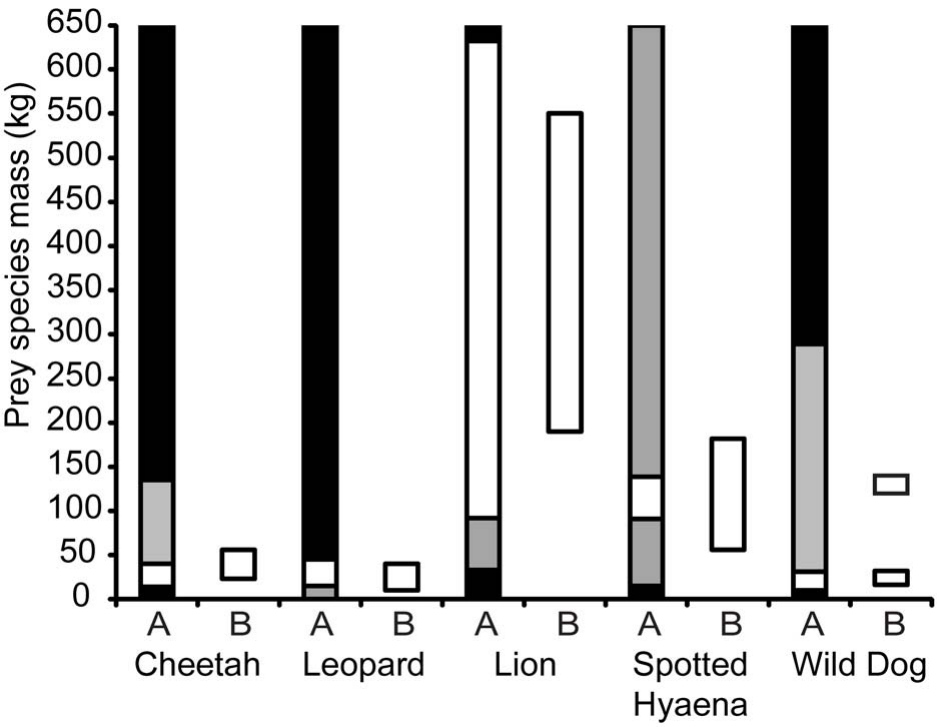
Prey weight preferences of the five large African carnivores. Weight ranges (below 650 kg) of prey species found to be preferred (white), killed relative to their abundance (grey) and avoided (black) by the five large African carnivores, determined (A) in this study and (B) in [5]–[9].

For cheetah, there are four significant changes in the relationship between prey species mass-rank and prey preference (AIC = 13.5, n = 46). These occur at a prey species mass-rank of 12.8, 21.3, 32.8 and 39.8, corresponding to a prey species mass of 14 kg, 40 kg, 135 kg and 319 kg, respectively (Fig. 2a). Prey species weighing 14 kg or less are avoided (J.I. = −0.62±0.14, *W* = 6, n = 14, *p*<0.01), prey species weighing between 14 kg and 40 kg are preferred (J.I. = 0.49±0.06, *W* = 393, n = 28, *p*<0.001), prey species weighing between 40 kg and 135 kg are killed relative to their abundance in the prey community (J.I. = −0.14±0.08, *W* = 123, n = 28, *p* = 0.07) and prey species weighing more than 135 kg are avoided (135 kg to 319 kg: J.I. = −0.54±0.07, *W* = 13, n = 28, *p*<0.001; >319 kg: J.I. = −0.91±0.04, *W* = 0, n = 26, *p*<0.001; Fig. 3).

For leopard, there are three significant changes in the relationship between prey species mass-rank and prey preference (AIC = 48.3, n = 48). These occur at a prey species mass-rank of 18.5, 28.0 and 41.8, corresponding to a prey species mass of 15 kg, 45 kg and 325 kg, respectively (Fig. 2b). Prey species weighing 15 kg or less are killed relative to their abundance (J.I. = −0.26±0.12, *t* = −2.07, d.f. = 15, *p* = 0.06), prey species weighing between 15 kg and 45 kg are preferred (J.I. = 0.55±0.06, *W* = 429, n = 29, *p*<0.001) and prey species weighing more than 45 kg are avoided (45 kg to 325 kg: J.I. = −0.50±0.07, *W* = 17, n = 29, *p*<0.001; >325 kg: J.I. = −0.84±0.08, *W* = 6, n = 28, *p*<0.001; Fig. 3).

For lion, there are four significant changes in the relationship between prey species mass-rank and prey preference (AIC = −24.9, n = 34). These occur at a prey species mass-rank of 5.4, 12.0, 19.6 and 30.4, corresponding to a prey species mass of 13 kg, 32 kg, 92 kg and 632 kg, respectively (Fig. 2c). Prey species weighing 32 kg or less are avoided (≤13 kg: J.I. = −0.81±0.16, *W* = 1, n = 10, *p*<0.01; 13 kg to 32 kg: J.I. = −0.63±0.07, *W* = 55, n = 42, *p*<0.001), prey species weighing between 32 kg and 92 kg are killed relative to their abundance (J.I. = −0.02±0.08, *W* = 449, n = 42, *p* = 0.98), prey species weighing between 92 kg and 632 kg are preferred (J.I. = 0.48±0.06, *W* = 966, n = 45, *p*<0.001) and prey species weighing more than 632 kg are avoided (J.I. = −0.69±0.16, *W* = 4, n = 10, *p* = 0.02; Fig. 3).

For spotted hyaena, there are three significant changes in the relationship between prey species mass-rank and prey preference (AIC = −4.2, n = 30). These occur at a prey species mass-rank of 6.2, 17.9 and 20.7, corresponding to a prey species mass of 15 kg, 91 kg and 139 kg, respectively (Fig. 2d). Prey species weighing 15 kg or less are avoided (J.I. = −0.40±0.11, *W* = 9, n = 13, *p* = 0.01), prey species weighing between 15 kg and 91 kg are consumed relative to their abundance (J.I. = −0.09±0.12, *t* = −0.76, d.f. = 19, *p* = 0.46), prey species weighing between 91 kg and 139 kg are preferred (J.I. = 0.27±0.08, *W* = 149, n = 18, *p*<0.01) and prey species weighing more than 139 kg are consumed relative to their abundance (J.I. = −0.04±0.12, *t* = −0.35, d.f. = 20, *p* = 0.73; Fig. 3).

For wild dog, there are three significant changes in the relationship between prey species mass-rank and prey preference (AIC = 26.4, n = 41). These occur at a prey species mass-rank of 7.0, 14.7 and 34.6, corresponding to a prey species mass of 10 kg, 31 kg and 289 kg, respectively (Fig. 2e). Prey species weighing 10 kg or less are avoided (J.I. = −0.50±0.19, *W* = 7, n = 11, *p* = 0.02), prey species weighing between 10 kg and 31 kg are preferred (J.I. = 0.35±0.09, *W* = 279, n = 25, *p*<0.01), prey species weighing between 31 kg and 289 kg are killed relative to their abundance (J.I. = −0.20±0.09, *W* = 88, n = 25, *p* = 0.05) and prey species weighing more than 289 kg are avoided (J.I. = −0.94±0.03, *W* = 0, n = 22, *p*<0.001; Fig. 3).

**Figure 4:**
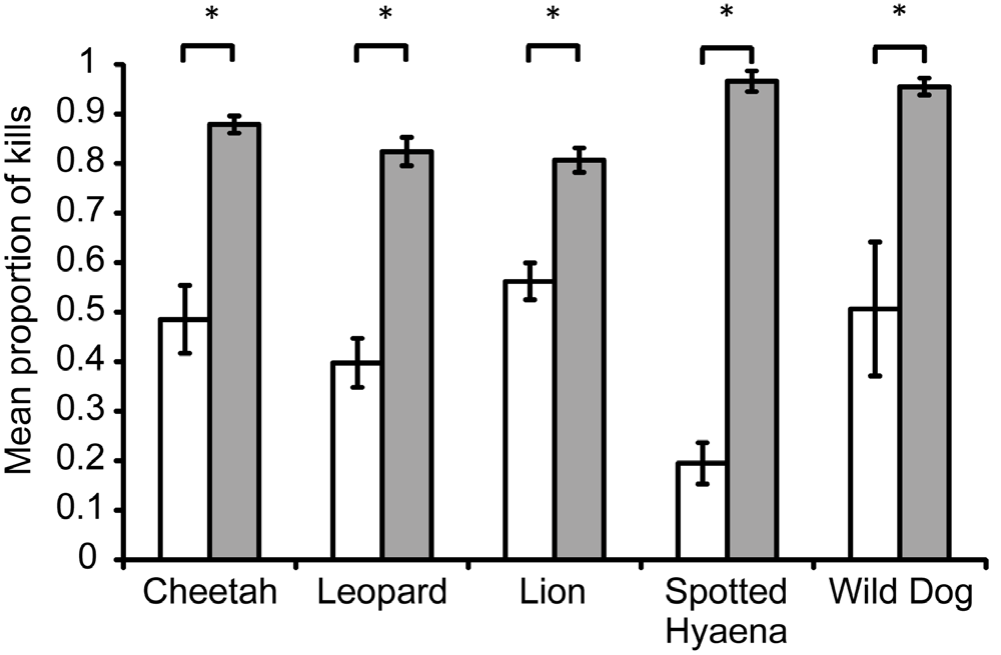
Mean (±SE) proportions of kills falling within the preferred and accessible weight ranges at test sites. The mean proportion of kills made by each of the five large African carnivores at test sites that fall within this study’s preferred (white) and accessible (grey) prey weight ranges. * - significant difference between the proportion of kills in the weight ranges preferred by and accessible to a carnivore.

The accessible prey weight ranges identified in this study account for, on average, more than 80% of each carnivore’s diet at test sites, with standard errors less than 4% (Fig. 4). The accessible prey weight ranges account for a significantly greater proportion of each carnivore’s diet than do the preferred prey weight ranges at test sites (cheetah: *t* = −6.37, d.f. = 13, *p*<0.001; leopard: *t* = −7.10, d.f. = 20, *p*<0.001; lion: *t* = −9.01, d.f. = 31, *p*<0.001; spotted hyaena: *t* = −22.72, d.f. = 6, *p*<0.001; wild dog: *t* = −3.26, d.f. = 6, *p* = 0.01; Fig. 4).

## Discussion

This study’s use of a segmented model to recalculate the prey weight preferences of the five large African carnivores is objective. It is based on statistically determined changes in prey preference, adhering to minimum species sample size requirements determined using simulations of known prey preference. In contrast, the previously used distance-weighted-least-squares method is subjective and based on a non-statistical, subjectively determined “most preferred” prey weight range [5]. The preferred prey weight ranges obtained using the objective approach generally support the previously determined weight ranges, particularly for cheetah and leopard. Some refinements are notable: this study finds the preferred weight range of lion to be 180 kg broader than that determined by [5]. In contrast, this study finds the preferred weight range of spotted hyaena to be 78 kg narrower than that determined by [7], though the weight range found to be accessible to spotted hyaena in this paper encompasses that found to be preferred by [7]. This study finds wild dog to prefer a single weight range, corresponding to the lower weight range found to be preferred by [9]. The additional, larger weight range found to be preferred by [9] is encompassed within the weight range found by this study to be accessible to wild dog. In addition, unlike the subjective method, the segmented model method identifies not only the weight range of prey preferred by each carnivore, but also the weight range of prey killed relative to their abundance, thereby identifying each carnivore’s accessible prey weight range. For all five large African carnivores, when tested across a diverse array of reserves in Africa, these accessible prey weight ranges accounted for over 80% of carnivore diet, with low variation across varied vegetation types and prey communities. This study’s novel segmented model approach therefore provides an accurate description of each carnivore’s prey weight spectrum.

Obtaining a broad-scale understanding of predator prey preference requires the use of data from multiple sites. Determining the accuracy of prey species abundances in such data is challenging. For this reason, this study utilizes data from a large sample of studies (Table S1), and measures preference as an average across these datasets. For a prey weight range to be found to be significantly preferred or avoided, it must be so across a large number of datasets. The value of this kind of analysis is that it is not biased by the results from one particular study. While reducing random bias from individual datasets, what is not eliminated by this form of analysis are biases that are likely to be inherent across all datasets. In dietary studies of large carnivores, a potential bias is the undercounting of small prey species in abundance measures. While we are unable to eliminate such a bias, we reiterate the argument made by [5] that the underestimation of the population size of small species is likely to be counteracted by the undercounting of the carcasses of these small species which are almost totally consumed. Such an assumption can be tested using the objective approach we present in this study when a sufficient number of datasets of better known accuracy become available.

While variables besides prey size will also influence prey preference at a local scale (e.g. climate and vegetation; prey age, sex, defense mechanisms and palatability), broad-scale models such as those presented here provide robust departure points against which hypotheses regarding predator-prey interactions at a finer scale can be formulated (e.g. [24], [25]). Similarly, while predator sex and hunting group size can also influence prey preferences [26], consistent with the objectives of the published prey weight ranges that we refined, we investigated carnivore diet from a population perspective whereby a population at each site contains both sexes and all hunting group compositions. We therefore expect our results to reflect the mean hunting group size across sites and the mean between sexes. Finally, it is important to interpret prey preferences of a single predator within the context of the large carnivore guild, since competition between predators can be an important determinant of prey preference [26]. This study provides insights into broad-scale trends in prey preferences, and does not account explicitly for intra-guild competition in the model. Since the majority of studies utilized in this analysis were from areas comprising complete or near-complete carnivore guilds, care must be taken when extrapolating the results of this study to areas lacking intact large carnivore guilds. As such, these broad scale preference ranges can be used as a departure point from which to test predictions regarding competitive interactions between the five large predators, with further refinements made to our understanding of preference as additional studies are conducted.

Preferred prey are likely to be those that a predator has evolved to optimally hunt [25]. However, when preferred prey occur at densities insufficient for optimal hunting, “prey switching” will occur whereby the predator will also kill other prey which it has not evolved to preferentially prey upon [27]. This is evident by the significantly greater proportion of each carnivore’s diet falling within the accessible prey weight range than within the preferred prey weight range, suggesting secondary prey constitutes an important part of each large carnivore’s diet. By determining both preferred and relatively killed prey species, this secondary prey is accounted for.

By additionally determining which prey are consistently avoided, this study quantified the upper prey mass threshold, above which the cost of prey capture outweighs the benefit of consuming these large-bodied prey species, thus making them generally inaccessible to the predator. Of the five large African carnivores, only spotted hyaena did not display this avoidance for prey above a certain mass-threshold. Spotted hyaena are known to scavenge, and therefore may consume prey larger than that which they could physically capture [28], [29]. As the spotted hyaena diet data used in this study were obtained from studies including scat analyses [7], scavenged prey will be included in diet descriptions and this most likely explains the spotted hyaena’s lack of avoidance of large-bodied prey. Given the shortage of spotted hyaena kill and prey abundance data available (21 studies amounting to 30 prey species), we were unable to exclude studies that utilized scats as the means of diet determination, in order to ensure a sufficient prey species sample size (Table 1). Carcasses available for scavenging will generally be those large enough not to be rapidly and entirely consumed by other carnivores. While scat studies were also used in the diet analysis of leopard [6], the leopard’s avoidance of prey weighing above 45 kg suggests that scavenging of larger animals is not influencing the preference findings for leopard, perhaps because leopard do not scavenge as regularly as spotted hyaena [30], [31].

While lion, cheetah and wild dog kill data did not include scat analyses, observation data can also include scavenged prey in instances where data are from observations of carnivores on a kill, as opposed to making a kill. This is unlikely to be an issue for the prey weight preference findings of cheetah and wild dog, who rarely scavenge [18], [32], but scavenging has been noted to comprise a proportion of the diet of lion in East Africa (16% of the diet according to [18]; see also [30], [33]). Considering that the majority of the lion kill data used in this study were from southern Africa, where scavenged prey is a minor component of lion diet [16], [34], scavenged prey are unlikely to be having a significant influence on the lion prey preference findings.

This study further quantified the lower prey mass-thresholds for each large carnivore, below which the energetic costs of seeking out and killing prey outweigh the energetic benefits of consuming these small-bodied individuals, resulting in prey below this threshold being consistently avoided. Such a threshold was evident for all carnivores except leopard, who hunt prey as small as birds and rodents [6]. The leopard’s stalking, solitary and highly adaptable hunting behaviour [6] therefore allows small prey to be an energetically viable hunting option. This is in contrast to predators who live and hunt, at least some of the time, in groups (e.g. lion, spotted hyaena, wild dog and male cheetah), and therefore display increased collective energy requirements which necessitate predation on larger prey [26], [28], [32], [35]. The solitary nature of leopard may also explain why this carnivore has the narrowest accessible prey weight range of the large African carnivores, despite having a similar body mass to cheetah and a larger body mass than wild dog [36]. Cursorial predators (e.g. cheetah and wild dog) rely on speed/stamina, as opposed to stealth to hunt and therefore may not be efficient at hunting very small animals whose anti-predator response is dependent on their ability to detect a predator before it detects them [37]. While this is supported for wild dog in the Kruger National Park, where steenbok flight frequently failed to elicit chases from wild dog [38], exceptions can arise when small prey occur in high densities which increase encounter rate and thus reduce the effort of actively pursuing small prey (e.g. [39]).

The prey preferences of large carnivores are a useful tool with which to predict the success of carnivore reintroductions based on the availability of prey within the preferred prey weight range [40]. Furthermore, an improved understanding of prey preference increases the accuracy with which we can predict, and therefore manage, the impact of predation on threatened species. For example, roan antelope *Hippotragus equinus* are within the weight range preferred by lion, but are usually protected from predation through their scarcity [25]. Management actions in the Kruger National Park resulted in increased habitat congruence between roan and the lion’s preferred prey species, causing roan population collapse as a result of increased predation [41]. Such a situation could have been predicted with knowledge of lion prey preferences. Similarly, our findings suggest that the vulnerable Cape mountain zebra *Equus zebra zebra* is within the preferred prey weight range of lion. This knowledge highlights the need to mitigate Cape mountain zebra predation by lion that have recently been reintroduced at the Karoo and Mountain Zebra National Parks [42]. This could be done by increasing the relative abundance of other preferred prey species to provide a buffer for the Cape mountain zebra, and by managing lion numbers carefully [4].

The prey preferences of the five large African carnivores have been used to develop predator-prey abundance models, intended to serve as a tool for estimating sustainable numbers for reintroduced carnivores [40]. Regardless of the accuracy with which prey species preferences are predicted (85% for lion: [43]), such predictions do not account for secondary prey. Estimates of sustainable carnivore population sizes based solely on preferred prey may therefore underestimate sustainable predator density in areas where preferred prey species are low in abundance. While this remains to be adequately tested, the estimate for a sustainable leopard population in a mountainous area where preferred prey species are scarce was 15% lower than the observed leopard density [19]. Since the accessible prey weight ranges identified in this study accurately and consistently account for a large proportion of carnivore diet, the abundance of prey in these weight ranges may prove to be an improved correlate of predator density, and therefore provide improved predictions of sustainable predator population numbers. This remains to be tested.

The segmented model method proposed by this study can be used to determine and compare the prey weight preferences of other predators, with the resolution of prey preference findings dependent on the sample size of prey species available (according to Table 1). For example, if a predator has a low number of prey species available to it, we would expect to have an 85% chance of detecting a single change in prey weight preference (if it exists) with just eight prey items. However, an 85% chance of detecting a second change would require considerably more (n = 21) prey species. This method and the sample size guidelines could be used to refine the subjective preferred prey weight range described for tiger *Panthera tigris* [44], as well as for calculating the tiger’s accessible prey weight range. The method has been used for snow leopard *Panthera uncia* [45], and data are currently being collected for multi-site prey preference analyses of dole *Cuon alpinus*, jaguar *Panthera onca*, coyote *Canis latrans*, puma *Puma concolor*, Eurasian lynx *Lynx lynx* and clouded leopard *Neofelis nebulosa* (Hayward pers comm).

Both the refined preferred prey weight ranges, and newly determined accessible prey weight ranges of the five large African carnivores contribute to our understanding of large carnivore prey spectrum and preference. These weight ranges should therefore prove useful for large carnivore conservation and management. Furthermore, these carnivores serve as an example of how the segmented approach can be used to assess the prey weight preferences and diet spectrum of other carnivore species.

## Supporting Information

Table S1 **Details of carnivore kill and prey abundance data used to calculate preferred and accessible prey weight ranges.** The sources, location and timing of African studies, and the number of kills recorded in each, used by [5]–[9], and used again in this paper, to calculate preferred prey weight ranges of cheetah, leopard, lion, spotted hyaena and wild dog.
(XLSX)

Table S2 **Prey species masses, mass-ranks and mean Jacob’s Index (J.I.) values used in segmented model analyses.**
(XLSX)

Table S3 **Details of carnivore kill and prey abundance data used to test preferred and accessible prey weight ranges.** The sources, location and timing of African studies, and the number of kills recorded in each, used to assess the mean percentage of the diet accounted for by the preferred and accessible prey weight ranges determined in this study.
(XLSX)

Code S1 **Code used to generated segmented linear relationships using the segmented generalized linear function in the segmented package** [21] **of the open source statistical program R (R Development Core Team 2012).**
(R) Click here for additional data file.

## References

[1] Ginsberg JR (2001) Setting priorities for carnivore conservation: what makes carnivores different? In: Gittleman JL, Funk SM, Macdonald DW, Wayne RK, editors. Carnivore Conservation. Cambridge: Cambridge University Press and The Zoological Society of London. 498–523.

[2] Carbone C, Gittleman JL (2002) A common rule for the scaling of carnivore density. Science 295: 2273–2276. 11910114 10.1126/science.1067994

[3] Karanth KU, Nichols JD, Kumar NS, Link WA, Hines JE (2004) Tigers and their prey: Predicting carnivore densities from prey abundance. Proc Natl Acad Sci U S A 101: 4854–4858. 15041746 10.1073/pnas.0306210101 PMC387338

[4] Hayward MW, O’Brien J, Hofmeyr M, Kerley GIH (2007) Testing Predictions of the Prey of Lion Derived From Modeled Prey Preferences. J Wildl Manage 71: 1567–1575.

[5] Hayward MW, Kerley GIH (2005) Prey preferences of the lion (*Panthera leo*). J Zool London 267: 309–322.

[6] Hayward MW, Henschel P, O’Brien J, Hofmeyr M, Balme G, et al (2006) Prey preferences of the leopard (*Panthera pardus*). J Zool London 270: 298–313.

[7] Hayward MW (2006) Prey preferences of the spotted hyaena (*Crocuta crocuta*) and degree of dietary overlap with the lion (*Panthera leo*). J Zool London 270: 606–614.

[8] Hayward MW, Hofmeyr M, O’Brien J, Kerley GIH, Henschel P, et al (2006) Prey preferences of the cheetah (*Acinonyx jubatus*) (Felidae: Carnivora): morphological limitations or the need to capture rapidly consumable prey before kleptoparasites arrive? J Zool London 270: 615–627.

[9] Hayward MW, O’Brien J, Hofmeyr M, Kerley GIH (2006) Prey preference of the African wild dog *Lycaon pictus* (Canidae: Carnivora): Ecological requirements for conservation. J Mammal 87: 1122–1131.

[10] Jacobs J (1974) Quantitaive measurements of food selection: a modification of the Forage Ratio and Ivlev’s Electivity Index. Oecologia 14: 413–417. 28308662 10.1007/BF00384581

[11] Fischhoff IR, Sundaresan SR, Cordingley J, Rubenstein DI (2007) Habitat use and movements of plains zebra (*Equus burchelli*) in response to predation danger from lions. Behav Ecol 18: 725–729.

[12] Wentworth JC, Tambling CJ, Kerley GIH (2011) Evidence for prey selection by spotted hyaena in the Eastern Cape, South Africa. Acta Theriol (Warsz) 56: 389–392.

[13] Dalerum F, Somers MJ, Kunkel KE, Cameron EZ (2008) The potential for large carnivores to act as biodiversity surrogates in southern Africa. Biodivers Conserv 17: 2939–2949.

[14] Meena V, Jhala YV, Chellam R, Pathak B (2011) Implications of diet composition of Asiatic lions for their conservation. J Zool London 284: 60–67.

[15] Ivlev VS (1961) Experimental ecology of the feeding of fishes. New Haven, CT.: Yale University Press.

[16] Owen-Smith N, Mills MGL (2008) Predator-prey size relationships in an African large-mammal food web. J Anim Ecol 77: 173–183. 18177336 10.1111/j.1365-2656.2007.01314.x

[17] Stuart CT, Stuart T (2000) Field guide to the larger mammals of Africa. Cape Town: Struik Publishers.

[18] Schaller GB (1972) The Serengeti Lion: A Study of Predator-Prey Relations. Chicago, Illinois, USA: The University of Chicago Press.

[19] Jooste E, Hayward MW, Pitman RT, Swanepoel LH (2013) Effect of prey mass and selection on predator carrying capacity estimates. Eur J Wildl Res 59: 487–494.

[20] Hudson DJ (1966) Fitting Segmented Curves Whose Join Points Have to Be Estimated. J Am Stat Assoc 61: 1097–1129.

[21] Muggeo V (2008) Segmented: an R package to fit regression models with broken-line relationships. Rnews 8: 20–25.

[22] Akaike H (1974) A new look at the statistical model identification. IEEE Trans Automat Contr 19: 716–723.

[23] Zar JH (1996) Biostatistical Analysis. 3rd ed. New Jersey: Prentice-Hall International Limited.

[24] Svengren H, Björklund M (2010) An Assessment of the Density of a Large Carnivore using a Non-Invasive Method Adapted for Pilot Studies. South African J Wildl Res 40: 121–129.

[25] Hayward MW (2011) Scarcity in the prey community yields anti-predator benefits. Acta Oecologica 37: 314–320.

[26] Radloff FGT, du Toit JT (2004) Large predators and their prey in a southern African savanna: a predator’s size determines its prey size range. J Anim Ecol 73: 410–423.

[27] Garrott RA, Bruggeman JE, Becker MS, Kalinowski ST, White PJ (2007) Evaluating prey switching in wolf-ungulate systems. Ecol Appl 17: 1588–1597. 17913125 10.1890/06-1439.1

[28] Kruuk H (1970) Interactions between populations of spotted hyaenas (*Crocuta crocuta* Erxleben) and their prey species. In: Watson A, editor. Animal populations in relation to their food resources. Oxford: Blackwell Scientific Publications. 359–374.

[29] Di Silvestre I, Novelli O, Bogliani G (2000) Feeding habits of the spotted hyaena in the Niokolo Koba National Park, Senegal. Afr J Ecol 38: 102–107.

[30] Kruuk H (1972) The Spotted Hyaena. A Study of Predation and Social Behaviour. Chicago: University of Chicago Press.

[31] Bertram BCB (1979) Serengeti predators and their social systems. In: Sinclair ARE, Norton-Griffiths M, editors. Serengeti: dynamics of an ecosystem. Chicago: University of Chicago Press. 221–285.

[32] Caro TM (1994) Cheetah of the Serengeti Plains: Group living in an asocial species. Chicago: University of Chicago Press.

[33] Cooper SM, Holekamp KE, Smale L (1999) A seasonal feast: long-term analysis of feeding behaviour in the spotted hyaena (*Crocuta crocuta*). Afr J Ecol 37: 149–160.

[34] Funston PJ, Mills MGL, Biggs HC (2001) Factors affecting the hunting success of male and female lions in the Kruger National Park. J Zool London 253: 419–431.

[35] Funston PJ, Mills MGL, Biggs HC, Richardson PRK (1998) Hunting by male lions: ecological influences and socioecological implications. Anim Behav 56: 1333–1345. 9933529 10.1006/anbe.1998.0884

[36] Skinner JD, Chimimba CT (2005) The Mammals of the Southern African Subregion. Cambridge: Cambridge University Press.

[37] Jarman PJ (1974) The Social Organisation of Antelope in Relation to Their Ecology. Behaviour 48: 215–267.

[38] Reich A (1981) The behavior and ecology of the African wild dog (*Lycaon pictus*) in the Kruger National Park. New Haven, CT.: Yale University Press.

[39] Woodroffe R, Lindsey PA, Romanach SS, Ole Ranah SMK (2007) African wild dogs (*Lycaon Pictus*) can subsist on small prey: Implications for conservation. J Mammal 88: 181–193.

[40] Hayward MW, O’Brien J, Kerley GIH (2007) Carrying capacity of large African predators: Predictions and tests. Biol Conserv 139: 219–229.

[41] Harrington R, Owen-Smith N, Viljoen PC, Biggs HC, Mason DR, et al (1999) Establishing the causes of the roan antelope decline in the Kruger National Park, South Africa. Biol Conserv 90: 69–78.

[42] Hrabar H, Kerley GIH (2013) Conservation goals fors the Cape mountain zebra *Equus zebra zebra*–security in numbers? Oryx 47: 403–409.

[43] Louw J, Funston P, Greeff A, Kloppers H (2012) The Applicability of Lion Prey Selection Models to Smaller Game Reserves in South Africa. South African J Wildl Res 42: 73–81.

[44] Hayward MW, Jędrzejewski W, Jêdrzejewska B (2012) Prey preferences of the tiger Panthera tigris. J Zool London 286: 221–231.

[45] Lyngdoh S, Shrotriya S, Goyal SP, Clements H, Hayward MW, et al (2014) Prey Preferences of the Snow Leopard (*Panthera uncia*): Regional Diet Specificity Holds Global Significance for Conservation. PLoS One 9: e88349. 24533080 10.1371/journal.pone.0088349 PMC3922817

